# The prodomain of caspase-3 regulates its own removal and caspase activation

**DOI:** 10.1038/s41420-019-0142-1

**Published:** 2019-01-28

**Authors:** Katelyn G. Ponder, Lawrence H. Boise

**Affiliations:** 1 Cancer Biology Graduate Program, Emory University, Atlanta, GA USA; 2 Hematology and Medical Oncology, Winship Cancer Institute of Emory University, Atlanta, GA USA

## Abstract

Caspase-3 is a cysteine–aspartic acid protease that cleaves cellular targets and executes cell death. Our current understanding is caspase-3 is activated by the cleavage of the interdomain linker and then subsequent cleavage of the N-terminal prodomain. However, previous reports have suggested that removal of the prodomain can result in the constitutive activation of caspase-3, although other studies have not observed this. To address this question in a more physiological setting, we developed an inducible doxycycline system to express a mutant form of caspase-3 that lacks the prodomain (∆28). We found that the removal of the prodomain renders the cells more susceptible to death signals, but the caspase is not constitutively active. To elucidate the regions of the prodomain that regulate activity, we created deletion constructs that remove 10 and 19 N-terminal amino acids. Surprisingly, removal of the first 10 amino acids renders caspase-3 inactive. Following serum withdrawal, the interdomain linker is cleaved, however, the remaining prodomain is not removed. Therefore, there is a specific amino acid or stretch of amino acids within the first 10 that are important for prodomain removal and caspase-3 function. We created different point mutations within the prodomain and found amino acid D9 is vital for caspase-3 function. We hypothesize that an initial cleavage event at D9 is required to allow cleavage at D28 that causes the complete removal of the prodomain allowing for full caspase activation. Together these findings demonstrate a previously unknown role of the prodomain in caspase activation.

## Introduction

Caspase-3 is a cysteine–aspartic acid protease that is best known for its enzymatic function at the end of the intrinsic apoptotic cascade. There are two classes of caspases that are involved in the process of apoptosis, initiator (e.g., caspase-8, -9) and executioner caspases (e.g., caspase-3, -7). Both groups are composed of a N-terminal prodomain, a large subunit (p20) and a smaller C-terminal subunit (p10)^1, 2^. Notably, the initiator caspases have a longer N-terminal prodomain, compared with the executioner caspases, and they are responsible for the initial cleavage of executioner caspases that leads to their activity^3, 4^. Executioner caspases are found within the cytoplasm as inactive zymogen dimers. Caspase-3, an executioner caspase, is held together as a dimer given the dimer interface is hydrophobic^5^. The dimer conformation also aids in the ability of initiator caspases to process the executioner caspases^6^.

The processing of the caspase-3 interdomain linker, found between the p20 and p10 domains, is completed by initiator caspase, caspase-9^7–9^. Once caspase-9 cleaves caspase-3 at the interdomain linker, caspase-3 undergoes a conformational change that exposes its active site found at amino acid C163. Previous studies have shown that caspase-3 undergoes two different cleavage events. The first, by caspase-9, within the interdomain linker and the second to remove the N-terminal prodomain^10^. Once activated, caspase-3 will cleave key structural proteins, cell cycle proteins, and DNase proteins, such as poly(ADP-ribose) polymerase, gelsolin, ICAD/DFF, and DNA-dependent kinase^11–13^. These cleavage events result in the blebbing and condensing of cells that ultimately leads to cell death^14^.

The apoptotic activity of caspase-3 is well characterized, but the regulation of this process is not fully understood. Previous studies demonstrated that the complete removal of the prodomain enhances apoptotic activity^15^. However, it is unknown whether this induction results in complete activation of caspase-3 or lowers the activation threshold. No studies have determined if the induction of activity is due to loss of full-length prodomain or a specific region within the prodomain. Additionally, no structural data of caspase-3 containing the prodomain have been determined. Therefore, we do not know where the prodomain is found in the inactive procaspase-3 enzyme. The prodomain is highly conserved suggesting it has a function (Fig. S1). Therefore, we undertook an investigation of the role of the prodomain in caspase-3 activation.

## Results

**Fig. 1:**
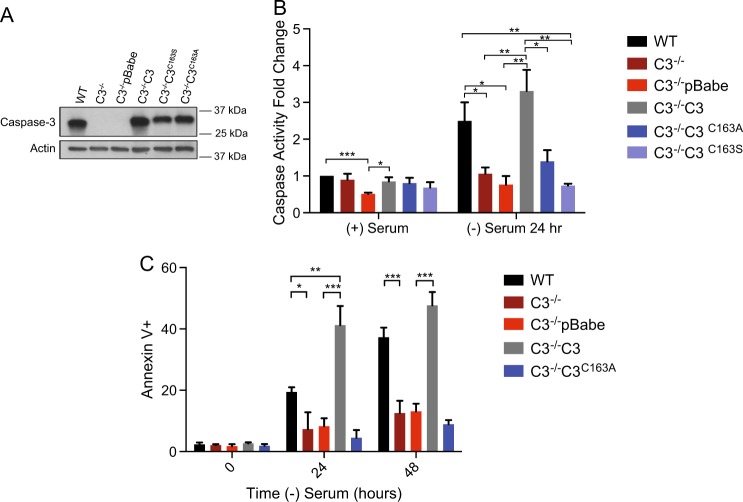
C3^−/−^C3 and C3^−/−^C3^C163A^ MEFs have apoptotic activity and caspase activity similar to that of WT and C3^−/−^pBabe, respectively. **a** Protein expression of caspase-3 and loading control actin of parental MEFs and stable cell lines expressing empty vector pBabe, full-length caspase-3 and two catalytically inactive forms of caspase-3, C163A and C163S. **b** Caspase-3 activity was measured using a caspase activity assay. Cells were grown in full serum as a control or serum starved for 24 h. A DEVD-chromphore substrate (DEVD-p-NA) was added to the protein lysis, and light emittance was measured at 405 nm. **c** Cells were serum starved for 0, 24, and 48 h to induce apoptosis. Apoptosis was determined by positive annexin V/propidium iodide (PI) and analyzed for cell death using flow cytometry. Data are presented as mean ±  SEM of at least three independent experiments. *p > 0.05, **p > 0.01, ***p > 0.001

To study the role of the prodomain in caspase-3 activation, we stably introduced caspase mutants into immortalized caspase-3-deficient mouse embryonic fibroblasts (MEFs). As can be seen in Fig. 1a, the level of expression of parental (C3^−/−^C3) or mutant forms of caspase-3 were similar to that observed in wild-type MEFs. Two different catalytically inactive forms of caspase-3, C163A and C163S, were expressed in caspase-3^−/−^ MEFs and used to demonstrate that the catalytic site at position 163 is essential for caspase-3 function. Introduction of full-length caspase-3 into the MEFs results in caspase activity (Fig. 1b) and the cells undergo apoptosis like the WT cells following serum withdrawal (Fig. 1c). However, the catalytically inactive forms, C163A and C163S, are inactive under these conditions (Fig. 1b) and do not induce cell death (Fig. 1c)^16^. These results confirm that this is a functional model to measure caspase regulation and function in a physiologic setting.

Previous studies have been conducted to determine the functional role of the prodomain of caspase-3. The studies conducted were performed using transient transfection, which could provide a stress signal to cells and confound apoptotic assays. Therefore, we sought to create a stable cell line that expresses a form of caspase-3 that lacks the 28 amino acid prodomain (∆28). We were able to create two stable cells lines, C3^−/−^C3∆28 and the catalytically inactive form C3^−/−^C3∆28^C163A^ (Fig. S2A). We used these cells to determine the amount of cell death and the caspase activity and found that, as expected, the C3^−/−^C3∆28^C163A^ cells did not undergo apoptosis and were not able to cleave a synthetic DEVD substrate. Unexpectedly, the C3^−/−^C3∆28 cells were able to cleave a synthetic substrate, but did not undergo cell death (Fig. S2B and S2C).

Given previous studies showed removal of the prodomain increases apoptotic activity we sought to determine if, when we created this stable cell line, we selected for cells that not only express our construct but also have a mutation or upregulation of another protein that could protect these cells from cell death. These cells have an increased expression of the x-linked inhibitor of apoptosis protein, XIAP (Fig. S2A). XIAP is an inhibitor of caspase-3 and the increased expression of XIAP is one potential explanation as to why the C3^−/−^C3∆28 MEFs do not die^17^. Given the stable expression of C3∆28 is lower than wildtype caspase-3 (Fig. S2A), we hypothesized that XIAP could be targeting C3∆28 to the proteasome^18, 19^. To test this hypothesis, we treated the cells with the protease inhibitor Carfilzomib, but did not see changes in the amount of cell death (Fig. S2D). Therefore, we believe that XIAP is working as a direct inhibitor of the caspase. Expressing caspase-3 and C3∆28 transiently was consistent with our findings as deletion of the prodomain resulted in significantly lower expression (Fig S2E). However, cell death was observed in a transient manner (Fig. S2F). Taken together these data suggest that removal of the prodomain does not result in a constitutively active caspase, however, the caspase has a lower activation threshold and these cells cannot tolerate its presence, even at physiologic levels. Therefore, removal of the prodomain makes caspase-3 easier to activate.

**Fig. 2:**
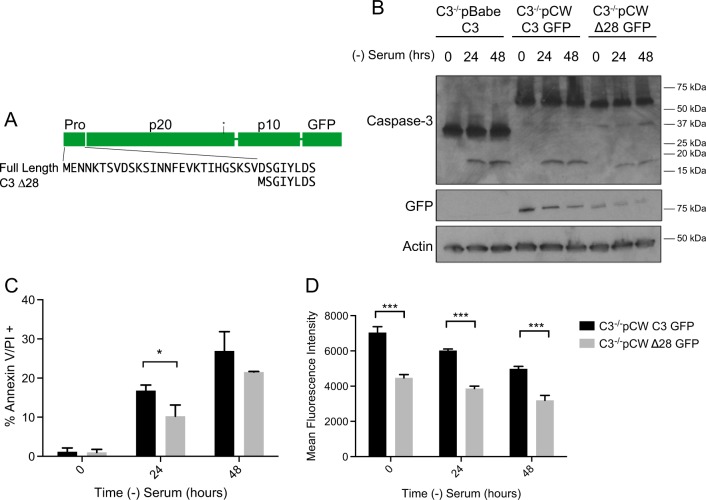
Complete removal of the prodomain results in apoptotic activity comparable to full-length caspase-3. **a** Diagram showing the C3 ∆28 construct lacks the 28 amino acid N-terminal prodomain. **b** Cells expressing a doxycycline inducible full-length caspase-3 tagged with GFP (C3 GFP) and caspase-3 lacking the prodomain (∆28 GFP) were stably expressed in caspase-3^−/−^ MEFs. Forty-eight hours post doxycycline treatment, cells were serum starved and collected at the indicated time points. Protein lysate was run on a western and probed for caspase-3, GFP and actin as a loading control. **c** Forty-eight hours post doxycycline treatment, cells were serum starved for the indicated times and cells were collected for flow cytometry analysis. Cell death was determined via positive annexin V/propidium iodide (PI) staining. **e** The mean fluorescence intensity (MFI) of the GFP signal was determined at the indicated time points. Data are presented as mean ± SEM of at least three independent experiments. *p > 0.05, **p > 0.01, ***p > 0.001

To be able to address the question of the role of the prodomain in caspase-3 apoptotic activity, without the stress associated with transient introduction, we turned to a doxycycline inducible system. We stably expressed two different doxycycline inducible plasmids in caspase-3^−/−^ MEFs, pCW C3 GFP and pCW C3∆28 GFP (Fig. 2a). We determined that after 48 h of 3 µg/mL doxycycline treatment, the cells express the constructs and the doxycycline treatment does not cause cell death (Fig. S3A and S3B). Following 48 h doxycycline treatment, serum withdrawal results in caspase activation with similar kinetics as the cells with constitutive caspase-3 expression (Fig. 2b). We next compared the amount of cell death in the C3 GFP and C3∆28 GFP MEFs. The cells were given doxycycline for 48 h and were serum starved for 0, 24, and 48 h. We analyzed only the cells that expressed the constructs by gating on the GFP-positive cell population. When comparing the C3 GFP and C3∆28 cells after 24 h, significantly more cell death was observed in the C3 GFP cells than the C3∆28 GFP cells, however, by 48 h the death in the two populations was similar (Fig. 2c). Comparing the mean fluorescence intensity (MFI) of these cells, the C3 GFP cells have a higher MFI than the C3∆28 GFP at all three time points (Fig. 2d). Thus, the likely explanation for the difference in cell death at 24 h is the disparity in protein expression. These data are consistent with C3∆28 not being constitutively active, but easier to activate. Thus, the prodomain appears to regulate caspase-3 activity.

**Fig. 3:**
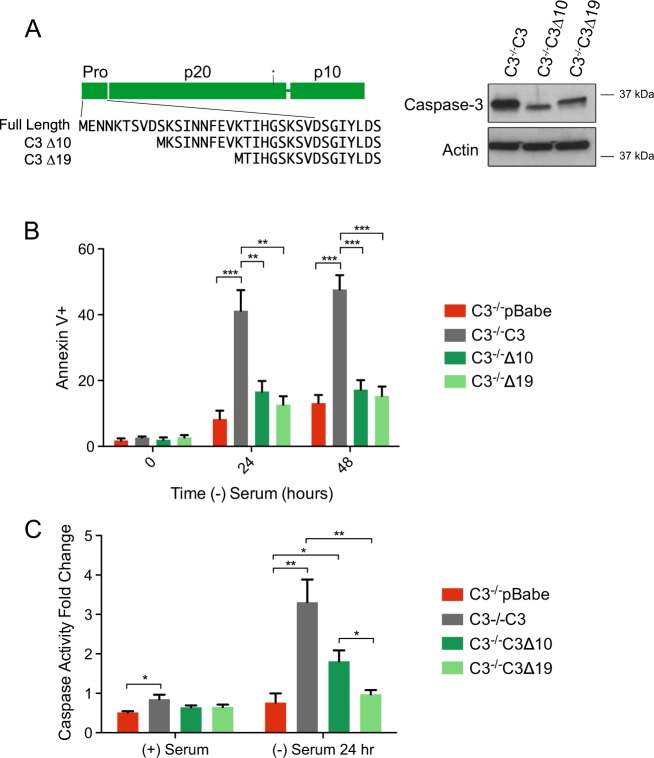
Removal of the first 10 amino acids decreases the apoptotic activity of caspase-3. **a** Deletion mutants were created by removing the first 10 (∆10) or first 19 (∆19) amino acids as shown in the left diagram. Protein expression was determined using western blot analysis for caspase-3 and actin as a loading control. **b** Deletion mutants were serum starved for the indicated times and subjected to cell death analysis via Annexin V/PI staining using flow cytometry. **c** A caspase-3 activity assay was used to determine the ability of the indicated cells to cleave a DEVD-chromphore substrate. Data are presented as mean ± SEM of at least three independent experiments. *p > 0.05, **p > 0.01, ***p > 0.001

The 28 amino acid prodomain of caspase-3 is highly conserved (Fig. S1A), therefore we sought to determine if there were specific regions within the prodomain that were important for its function in regulating caspase-3 activity. We initially generated two truncation mutants of the prodomain and expressed them stably in caspase-3^−/−^ MEFs (Fig. 3a). We removed the first 10 N-terminal amino acids (∆10) and the first 19 (∆19). Surprisingly, and in contrast to deletion of the full prodomain, neither truncation mutant was able to restore serum withdrawal induced apoptosis (Fig. 3b). Consistent with this phenotype, these deletions resulted in complete loss (C3∆19) or reduced (C3∆10, *p* = 0.058 compared with C3^−/−^C3) caspase activity upon serum withdrawal (Fig. 3c). Together these data suggest that the prodomain is removed because it contains a region that negatively regulates caspase-3 activity following caspase-9-mediated removal of the interdomain linker. Based on these findings, it also appears that the removal of the prodomain requires the first 10 amino acids.

**Fig. 4:**
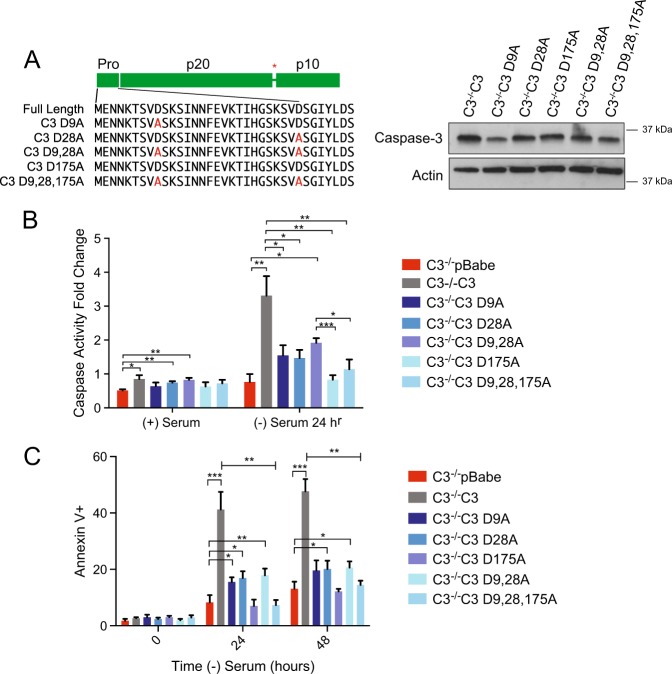
Mutating amino acid D9 to A9 decreases the apoptotic activity of caspase-3. **a** Point mutations within the prodomain and the interdomain linker were created and indicated in red. Caspase-3 protein expression was determined by western blot analysis. Actin was used as a loading control. **b** A caspase-3 activity assay was used on the indicated cell lines to determine the ability to cleave a DEVD-chromphore substrate. **c** The caspase-3 point mutation cells were subjected to cell death analysis via Annexin V/PI staining using flow cytometry. Data are presented as mean ± SEM of at least three independent experiments. *p > 0.05, **p > 0.01, ***p > 0.001

To elucidate the important amino acids within the prodomain for apoptotic regulation, we created various point mutations within the prodomain. Previous studies have demonstrated that mutating D9, D28, and D175 results in an uncleavable caspase^20^, therefore we focused on the role of D9 and D28 in caspase activity and induction of apoptosis following serum withdrawal. We created the single mutations, D9A, D28A, and D175A as well as double and triple mutations and stably expressed them in caspase-3^−/−^ MEFs (Fig. 4a). Consistent with previous findings, mutating all three sites results in a caspase that is not activated by serum withdrawal and does not induce apoptosis (Fig. 4b, c). However, mutated individually we found that the cells have significantly less caspase activation and do not undergo apoptosis (Fig. 4b, c). Interestingly, loss of D9 or D28 resulted in nearly identical changes in caspase activity and induction of apoptosis. This is consistent with the possibility that the prodomain must be cleaved at D9 prior to complete removal through cleavage at D28. Therefore, we next determined how the mutations alter caspase activation.

**Fig. 5:**
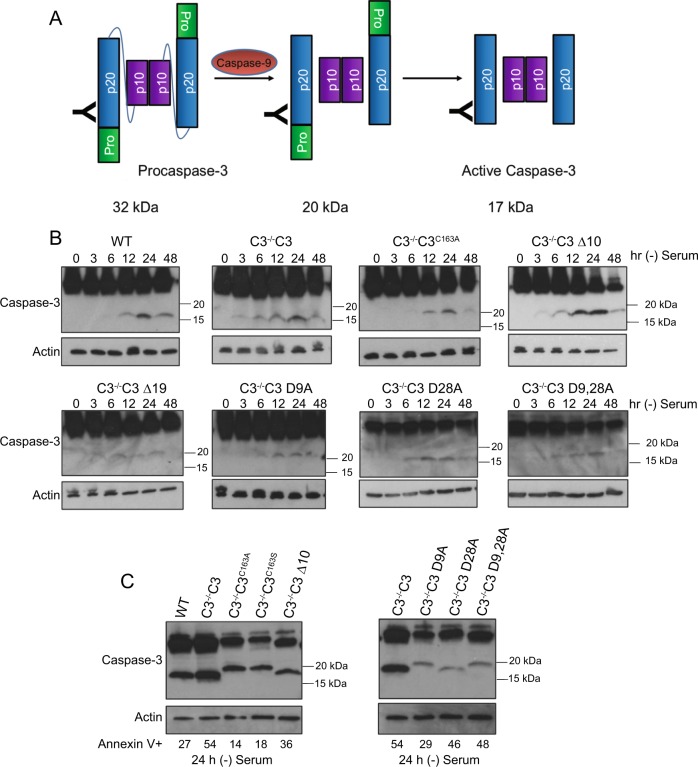
C3^−/−^C3^C163A^, C3^−/−^C3 ∆10, C3^−/−^C3 ∆19, and C3^−/−^C3 D9A are unable to remove the prodomain. **a** Diagram showing the resulting molecular weight bands, detected from an anti-caspase-3 antibody that binds to the p20 domain, resulting from cleavage of the interdomain linker and the prodomain. **b** Cell lines were serum starved and lysates collected at the times indicated. Western blot analysis was conducted to determine procaspase-3 cleavage. **c** Samples serum starved for 24 h were run on the same gel for direct comparison. Actin was used as a loading control

We directly assessed caspase-3 activation following serum starvation by measuring caspase-3 cleavage via western blot analysis. When caspase-3 is not cleaved it has a molecular weight of 32 kDa, which is detected on a western blot by a primary antibody that binds to the p20 domain. When the interdomain linker is cleaved and the prodomain is removed, resulting in the mature p20 fragment, the antibody detects a peptide at 17 kDa, but if the prodomain is not removed the fragment will run at 20 kDa (Fig. 5a). Blocking the interdomain cleavage through mutation of D175 results in complete loss of procaspase-3 processing following serum withdrawal (Fig. S4). Detection of cleaved caspase-3 is highest after 24 h in all other samples (Fig. 5b). Twenty-four hours of serum starvation resulted in the generation of the mature 17 kDa p20 fragment in C3^−/−^C3 cells. However, when the active site is mutated there is a shift in the mobility of the fragment to a molecular weight of 20 kDa, indicating that the prodomain is not removed (Fig. 5c). This is consistent with the model that the active site at C163 is responsible for removal of the prodomain. Interestingly deletion of the first 10 amino acids or mutation of D9 have no effect on interdomain cleavage, yet prevents the full maturation of the p20 domain. The fragment is the same size as the D9,28A mutant following serum withdrawal suggests that D9 is necessary for cleavage of D28. Consistent with this finding, the D28A mutant migrates faster than the D9,28A mutant suggesting that D9 is cleaved in the prodomain.

**Fig. 6:**
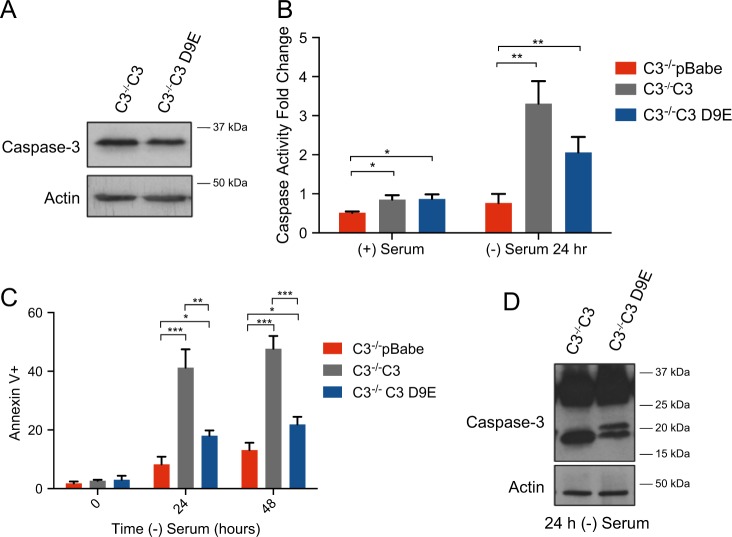
C3 D9E is inefficiently cleaved resulting in decreased caspase activation and cell death. **a** Expression of cells stably expressing C3 and C3 D9E. **b** A caspase-3 activity assay was used to determine the ability to cleave a DEVD-chromphore substrate. **c** Cell death was determined using Annexin V/PI staining and flow cytometry. **d** Cells were serum starved for 24 h and the cleavage pattern of caspase-3 was determined using western blot analysis. Actin was used as a loading control. Data are presented as mean ± SEM of at least three independent experiments. *p > 0.05, **p > 0.01, ***p > 0.001

Given the importance of amino acid D9 in prodomain removal, we wanted to further investigate the importance of cleavage at this site. The fact that D28A-cleaved product migrates faster than D9A-cleaved product supports a cleavage event at D9. We stably expressed a C3 D9E construct into caspase-3^−/−^ MEFs (Fig. 6a). The mutation of aspartic acid to glutamic acid results in a site that can still be cleaved by the caspase, albeit less effectively^21^.This mutation still supported significant caspase activity following serum withdrawal. However, there was only a minimal but significant increase in cell death compared with empty vector controls and significantly less death than observed in cells where WT caspase-3 was introduced (Fig. 6b, c, respectively). C3^−/−^C3 D9E MEFs were able to remove the prodomain, but inefficiently as indicated by the presence of bands at 17 and 20 kDa (Fig. 6d). These data support the hypothesis that there is a cleavage event at D9 that is required for full removal of the prodomain and full caspase-3 activation through cleavage at D28.

## Discussion

The apoptotic function of caspase-3 has been well characterized, but the mechanism by which procaspase-3 becomes active caspase-3 is not fully understood. Previous studies demonstrated in a cell-free system that there are two cleavage events to form the p17 and p12 subunits^10^. While one study concluded that removal of the prodomain resulted in a constitutively active caspase-3^15^, others have demonstrated that the prodomain does not influence apoptotic activity^22^. These studies expressed caspase-3 transiently. We utilized an inducible system to express C3∆28 because the stress from transiently expressing caspase-3 could be enough to activate caspase-3. Given that, at 48 h post doxycycline induction, the cells are expressing C3∆28 GFP at the same protein level as C3 GFP and there is no apoptosis occurring (Fig. 2b, c), we conclude that removal of the prodomain does not result in constitutive activation of caspase-3. Cleavage at the interdomain linker is still required. However, removal of the prodomain may allow for more efficient activation of caspase-3, suggesting that the prodomain plays a regulatory role in caspase-3 activation.

Indeed, our findings demonstrate that, in contrast to deleting the full prodomain, if one deletes part of the prodomain, the caspase cannot be efficiently activated. This suggests that a region within the prodomain is negatively regulating activation of caspase-3 following cleavage by caspase-9. Since the deletion of the first 19 residues resulted in a complete loss of function while the deletion of the first 10 residues retained activity, we propose that the negative regulatory region is located between residues 20 and 27. Deletion of the full prodomain removes this negative regulatory region. The discrepancy between the ∆10 and ∆19 activity suggested that loss of the first 10 amino acids could somehow facilitate activation. Therefore, we focused on the possibility that this region needs to be cleaved for activation.

There are two caspase-specific cleavage sites within the prodomain of caspase-3. Previous studies have shown, using recombinant caspase-3 or conducting experiments in vitro using cytosolic extracts, that there is a rapid cleavage event at D9 followed by a slower cleavage at D28^10, 22, 23^. Furthermore, an uncleavable form of procaspase-3 (D9A, D28A, and D175A), has been shown to have a lower catalytic efficiency^20^. To determine if these sites are important in a physiological setting, we tested the activity of MEFs expressing different point mutations within the prodomain. Although previous work had shown that there are cleavage events at D9 and D28, we found that mutating just D9 was sufficient to block activation and cell death.

These findings raise some questions about the regulation of caspase activation. Why are two cleavage events required to remove the prodomain? Our data demonstrate that the cleavage at D9 is required for cleavage at D28. One possible explanation is that recognition and binding of the D9 site orients the prodomain for cleavage at D28. This would imply that binding of the prodomain at D28 in the caspase active site is not efficient on its own. Unfortunately, the prodomain was not visible in the structure of procaspase-3, thus one can only speculate on its ability to bind the active site. Regardless if the D9 recognition is only for orientation, then cleavage at the site may not be necessary. Our findings suggest this is not the case, as mutation of D9 to glutamic acid resulted in a hypomorphic allele (Fig. 6d). This is consistent with caspases being able to cleave after glutamic acid at a lower efficiency than aspartic acid^21^. This suggests that in addition to orienting the prodomain for removal, cleavage at D9 must also be a regulatory event. This may also explain why the ∆10 construct displayed caspase activity following serum withdrawal. This suggests that additional negative regulatory elements may be destroyed by cleaving the prodomain at D9.

An interesting observation in our study is caspase cleavage and activity do not always correlate with cell death. This is not surprising for caspase cleavage and activity, as the initial cleavage event is not a measure of caspase-3 activity. This is really a measure of caspase-9 activity. However, it is surprising that the ∆10 construct and the D9E construct display significant caspase activity with little to no change in cell death. We hypothesize that this discrepancy is due to the requirements for demonstrating activity in these assays. Caspase activity is measured using a small four amino acid substrate in a cell lysate, while cell death requires cleavage of over 100 proteins substrates in whole cells. The activity assay would likely tolerate changes to the caspase structure due to deleting 10 amino acid residues or the D9E mutation while recognition of multiple protein substrates may be inhibited.

Finally, while our studies are limited to caspase-3, it is possible that a similar mechanism of regulation exists for the other main effector, caspase-7. The overall conservation of these prodomains is low, although this is primarily due to differences in the overall size of the prodomains. The caspase-7 prodomain is over twice the length of the prodomain of caspase-3. This may be due to other functions that have been attributed to the caspase-7 prodomain^24^. However, the most conserved region between the prodomains is centered around the D9 cleavage site. Further studies are required to test this possibility.

## Materials and methods

### Cell culture

Immortalized mouse embryonic fibroblasts (MEFs) were cultured as previously described^16^. ΦNX-Ecotropic packaging cell lines were grown in DMEM medium supplemented with 10% FBS, 1% penicillin–streptomycin, 1% l-Glutamine, 1% non-essential amino acids, and 1% sodium pyruvate.

### Cloning of plasmids

Primers were designed for the DNA sequence of interest, and polymerase chain reaction was performed using Taq PCR Kit (New England Biosystems, Waltham, MA, USA). After amplified target DNA was run on 1% agarose DNA gel, the DNA was isolated using the GFX^TM^ PCR DNA and Gel Band Purification Kit (GE Healthcare, Chicago, IL, USA). Isolated DNA and empty vector, pBabe, were digested and the ligation reaction was conducted using the DNA insert ligation kit (Thermo Fisher Scientific, Waltham, MA, USA) (Table S1).

### Site-directed mutagenesis

Primers for the indicated mutations were designed and mutagenesis was completed using the Quick Change XL Site-Directed Mutagenesis kit (New England Biosystems, Waltham, MA, USA) (Table S1).

### Retroviral transduction

ΦNX-Ecotropic packaging cells were transfected with a plasmid (pBabe-puro, C3 pBabe-puro, C3^C163A^ pBabe-puro, C3^C163S^ pBabe-puro, C3 ∆28 pBabe-puro, C3 ∆28^C163A^ pBabe-puro, C3 ∆10 pBabe-puro, C3 ∆19 pBabe-puro, C3 D9E pBabe-puro, C3 D9A pBabe-puro, C3 D9,28A pBabe-puro, C3 D175A pBabe-puro, C3 D9,28,175A pBabe-puro) using Lipofectamine (Invitrogen, Carlsbad, CA, USA). Target MEFs were then infected with retroviral supernatants using Polybrene Infection/Transfection Reagent (Millipore, Burlington, MA, USA). Two rounds of infection within a 36-h period was conducted. After 36 h, viral supernatants were removed from the target cells and replaced with fresh medium for 24 h and then were selected with 2.5 μg/ml puromycin (Sigma, St. Louis, MO, USA).

### Lentiviral transduction

In total, 293T cells were transfected with pHRCMV8.2 R, CMV-VSVG and either C3-GFP pCW-puro or C3∆28 pCW-puro using Lipofectamine (Invitrogen, Carlsbad, CA, USA). Target MEFs were infected as described above.

### Cell death analysis

In total, 0.25 × 10^6^ cells were seeded in six-well plates and allowed to grow overnight. Complete medium was replaced with serum-free medium to induce apoptosis. Cells were collected at 0, 24, and 48 h and stained with annexin V-FITC (BioVision 1001–1000, Milpitas, CA, USA) and propidium iodide (2 μg/ml Sigma, St. Louis, MO, USA). Cells were then measured with a BD FACSCanto II as described previously^25^. Data were analyzed using FlowJo (TreeStar, Ashland, OR, USA) software.

### Colometric caspase-3 activity assay

In total, 5 × 10^4^ cells were seeded in a 10-cm dish and allowed to grow for 24 h. Media was removed and replaced with either serum-free DMEM or complete DMEM. Cells were collected after 24 h. Caspase activity was determined using the Caspase-3 Assay Kit (Colorimetric) (Abcam ab39401, Cambridge, UK).

### Caspase-3 activation

In total, 0.25 × 10^6^ cells were seeded in six-well plates and allowed to grow overnight. Cells were serum starved with DMEM media lacking fetal bovine serum. After 0, 3, 6, 12, 24, and 48 h, cells were collected and lysed. Western blotting was performed as previously described^26^. Primary antibodies are the following: rabbit anti-caspase-3 (Cell Signaling, Danvers, MA, USA) and mouse anti-actin (Sigma, St. Louis, MO, USA).

### Immunoblotting

Western blotting was performed as previously described^26^. Primary antibodies: rabbit anti-caspase-3 (Cell Signaling, Danvers, MA, USA), XIAP (BD Biosciences, Franklin Lakes, NJ, USA) and mouse anti-actin (Sigma. St. Louis, MO, USA). Secondary antibodies are the following: horseradish peroxidase-conjugated sheep anti-mouse and horseradish peroxidase-conjugated donkey anti-rabbit (Amersham, Little Chalfont, UK). Proteins were detected by chemiluminescence (Amersham, Little Chalfont, UK).

## Supplementary information

Supplmental Figures
