# Supplementary material for: The prodomain of caspase-3 regulates its own removal and caspase activation
Source: Cell Death Discov. 2019 Jan 28;5:56. doi: 10.1038/s41420-019-0142-1 (PMC6349851; doi:10.1038/s41420-019-0142-1)
Supplement: Supplementary file 1 — Supplmental Figures [file 41420_2019_142_MOESM1_ESM.pdf]

A

B

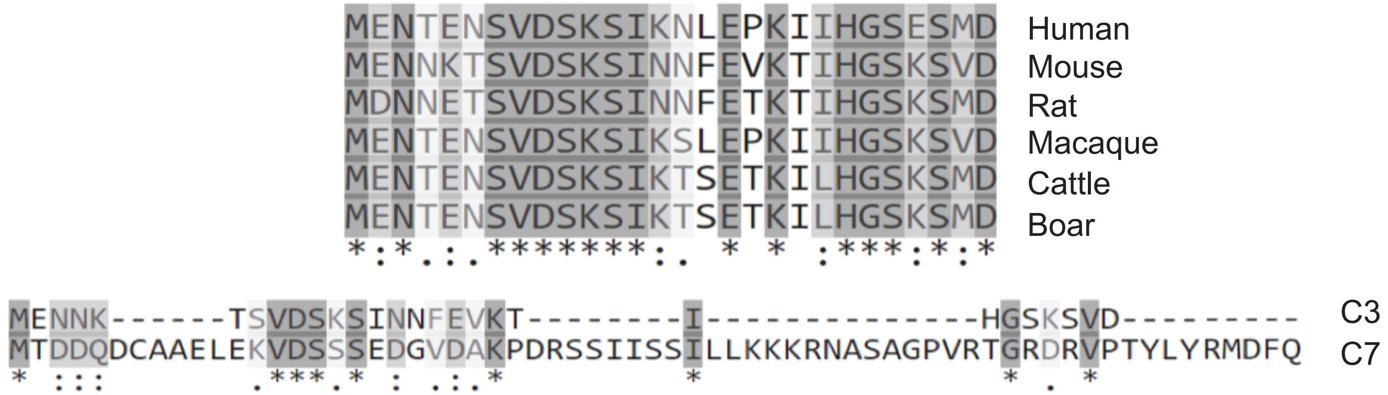

Supplemental Figure 1. The prodomain of caspase-3 is conserved, yet distinct from caspase-7. A. The percent identity of the prodomain of procaspase-3 between the 6 different species shown is 57.1%. The shaded grey areas highlight the identity. B. The prodomains of mouse procaspase-3 and -7 were analyzed in ScanProsite and only have an overall identity of 13.8%. The shaded grey areas highlight the identity between the prodomains.

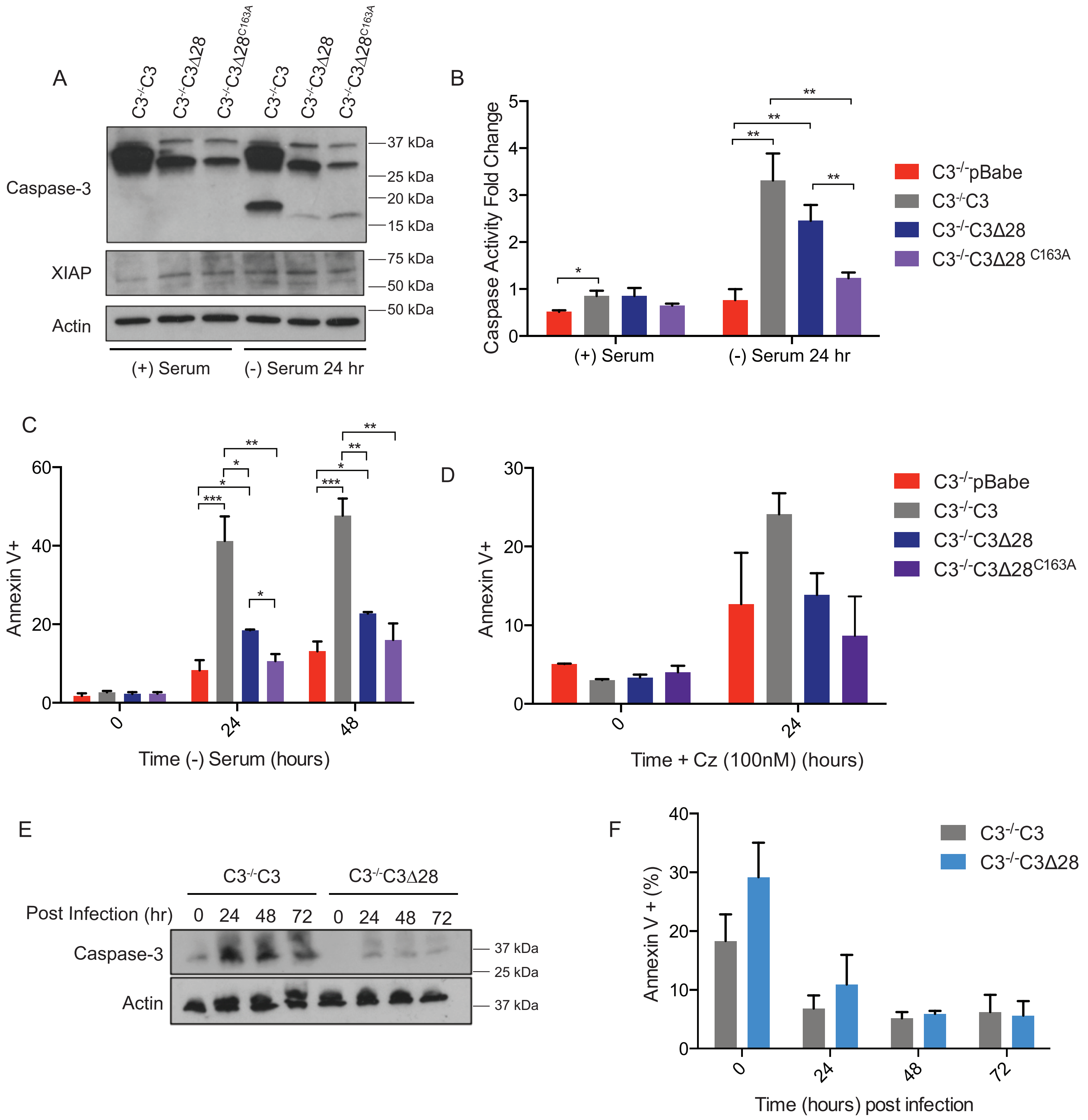

Supplemental Figure 2. Generation of stable C3Δ28 constructs and transient expression of C3Δ28 in caspase-3$^{-/-}$ MEFs. A. Cells stably expressing C3Δ28 and C3Δ28^{C163A} were generated and were used to determine the amount of cleaved caspase-3 and XIAP. B and C. Caspase activity and cell death were determined using a caspase-3 assay and annexin V/PI flow cytometry, respectively. D. Cells were treated with the proteasome inhibitor Carfilzomib (Cz) (100 nM) for the times indicated and cell death was determiend using annexin V/PI flow cytometry. (N=2) E. Caspase-3$^{-/-}$ MEFs were transiently infected with full length caspase-3 (C3) or caspase-3 lacking the prodomain (C3Δ28). Protein expression of C3 of C3Δ28 was determined 0-72 hours post infection. F. Cells were collected at the indicated time points and cell viability was determined by annexin V/ propidium iodide (PI) and analyzed for cell death using flow cytometry. Data are presented as mean +/- SEM of at least 3 independent experiments. $^{*}p > 0.05$, $^{**}p > 0.01$, $^{***}p > 0.001$

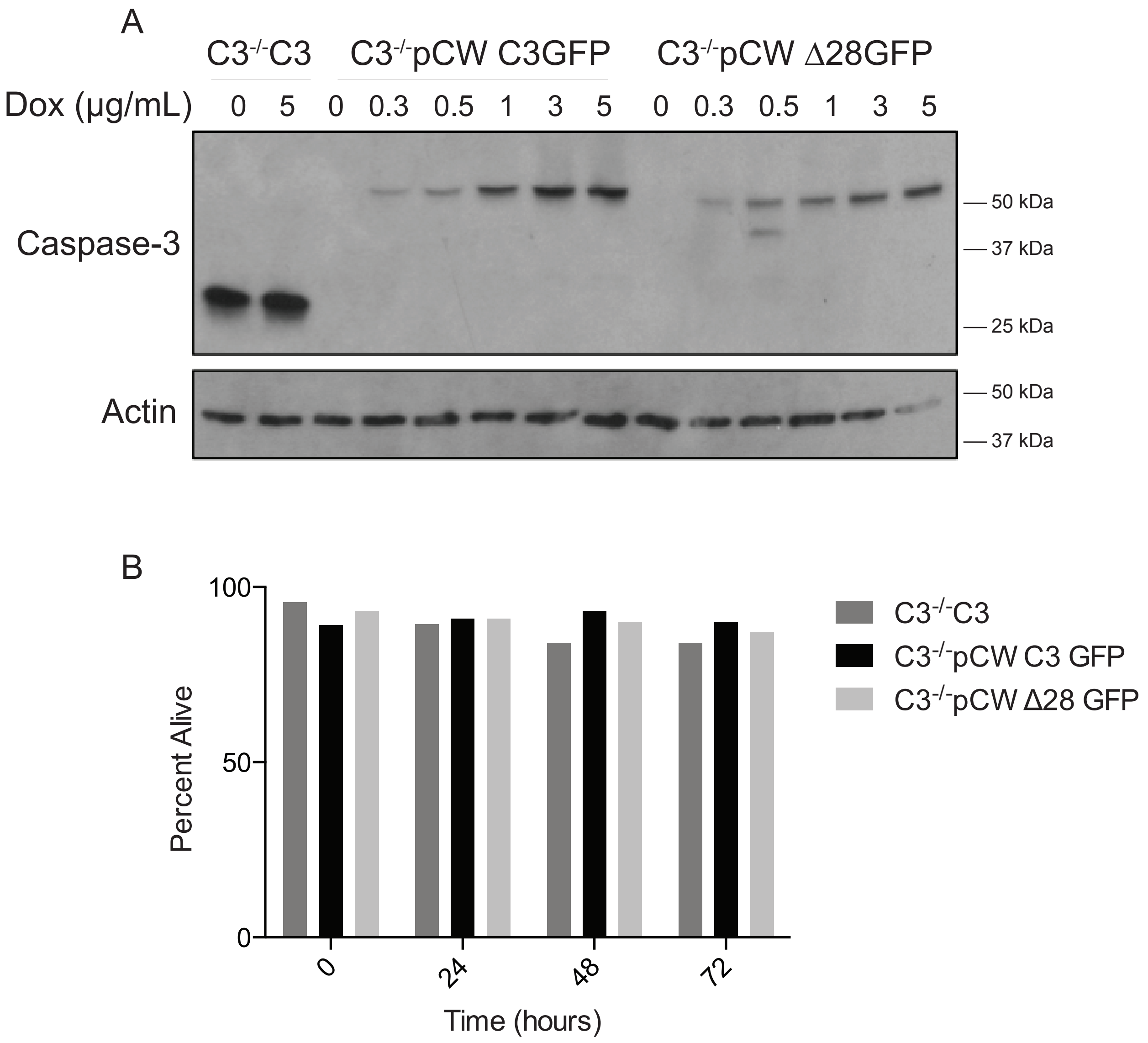

Supplemental Figure 3. Treatment with 3 μg/mL of doxycycline for 48 hours is sufficient for expression of C3 GFP and Δ28 GFP. Caspase-3$^{-/-}$ MEFs stably expressing pCW C3 GFP and pCW Δ28 GFP were generated. A. The indicated concentrations of doxycycline (Dox) were added to induce the expression of the plasmids as shown by western blot. B. Cell viability was determined after 3 μg/mL doxycycline treatment for the indicated time points using annexin V/PI staining. C3$^{-/-}$pBabe C3 MEFs were used to determine the effect of doxycycline on a non-inducible cell line.

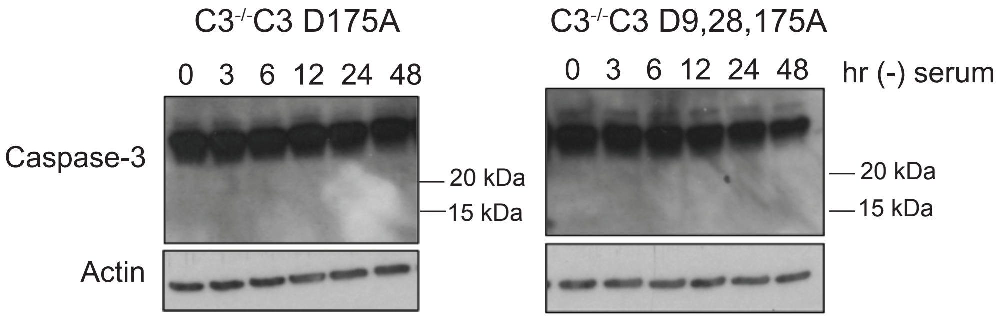

Supplemental Figure 4. Mutations D175A and D9,28,175A result in the inability of the interdomain linker to be cleaved. C3$^{-/-}$C3 D175A and C3$^{-/-}$C3 D9,28,175A MEFs were serum starved for the indicated times and the proteins lysates were analyzed via western blot. Actin was used as a loading control.

A

Plasmid	5' Primer
pBabe C3^{C163A}	tcatcattcaggccgcccggggtacggagc
pBabe C3 D9A	acaacaaaacctcagtggcctcaaaatccattaataattt
pBabe C3 D28A	gggagcaagtcagtggcctctgggatctatctg
pBabe C3 D175A	tgtggcattgagacagccagtggtgactgatgag
pBabe C3 D9E	acaacaaaacctcagtggagtcaaaatccattaataattt
pBabe C3 Δ10	cgccggccggatccgtgaccatgaaatccattaataat
pBabe C3 Δ19	cgccggccggatccgtgaccatgaccatacatgggagcaagtc

B

Plasmid	Template DNA	5' Primer	3' Primer
pBabe C3 Δ28	pBabe C3	taggcggattgtctgggatctatctggac	tggcccagctgctagtgataaaagtacagtt
pBabe Flag C3	pBabe C3	tggcccagctgctagtgataaaagtacagtt	tggcccagctgctagtgataaaagtacagtt
pBabe Flag C3 D175A	pBabe C3 D175A	tggcccagctgctagtgataaaagtacagtt	tggcccagctgctagtgataaaagtacagtt
pBabeC3 Δ28 GFP	pBabe C3 GFP	ggtggtaagcttatgtcgaagggcgaggagc	ggtggttagctactacctgtacagctcctcgtcc
pCW C3 GFP	pBabe C3 GFP	taggcgctagcatggagaacaacacctcagtg	gaagcggatccttacttgtacagctcgtcc
pCWC3 Δ28 GFP	pBabeC3 Δ28 GFP	taggcgctagcatgtctgggatctatctggac	gaagcggatccttacttgtacagctcgtcc

Supplemental Table 1. Primers used to generate caspase-3 mutation plasmids. A. The indicated plasmids were generated via site directed mutagenesis using the corresponding primers. B. The indicated plasmids were generated by PCR based cloning with the indicated template DNA and primers.
